# Score for the Survival Probability of Patients With Orbital Rhabdomyosarcoma After Surgery: A Long-Term and Large Cohort Study

**DOI:** 10.3389/fonc.2020.01590

**Published:** 2020-08-27

**Authors:** Yu Zhang, Chaobin He, Yu Lian, Huiming Xiao

**Affiliations:** ^1^State Key Laboratory of Ophthalmology, Zhongshan Ophthalmic Center, Sun Yat-sen University, Guangzhou, China; ^2^State Key Laboratory of Oncology in South China, Department of Pancreaobiliary Surgery, Collaborative Innovation Center for Cancer Medicine, Sun Yat-sen University Cancer Center, Guangzhou, China

**Keywords:** orbit rhabdomyosarcoma, overall survival, cancer-specific mortality, nomogram, prognosis

## Abstract

Orbital rhabdomyosarcoma (RMS) is a relatively rare primary malignancy occurring in children. The objective of this study was to evaluate the cumulative incidence of cancer-specific death and competing risk of death among RMS patients after surgery and to build nomograms to predict overall survival (OS) and cancer-specific survival (CSS) based on a large population-based cohort. The records of 217 patients who were pathologically diagnosed with an orbital RMS between 1973 and 2015 from the Surveillance, Epidemiology, and End Results (SEER) database were retrospectively analyzed. The 10-, 20-, and 40-years OS rates and cancer-specific mortality were 82.5, 72.2, and 48.9%, respectively, and 14.8, 21.7, and 21.7%, respectively. The established nomograms were well-calibrated and validated, with a concordance index (C-index) of 0.901 and 0.944 for OS prediction, 0.923 and 0.904, for CSS prediction in the training and validation cohorts, respectively. The values of area under the receiver operating characteristic curve (AUC) for 10-, 20-, and 40-years OS and CSS prediction were 0.908, 0.826, and 0.847, and 0.924, 0.863, and 0.863, respectively. The established nomogram showed relatively good performances and could be convenient individualized predictive tools for prognostic prediction in RMS patients.

## Introduction

Rhabdomyosarcoma (RMS) represents a malignant tumor of skeletal muscle origin, accounting for 5–10% of childhood cancers and more than 50% of pediatric soft tissue sarcomas (1). In about 35% of cases, the head and neck are the main sites of RMSs, with most tumors located in the orbit, which contributes to 10% of all sites of RMSs (2). Orbital RMSs are the most common primary malignancy occurring in children, and patients with orbital RMSs have a relatively good prognosis after multimodality therapy, including surgery, chemotherapy, and radiotherapy (3, 4). Surgery plays an extremely important role in the treatment of orbital RMSs because the removal of most of the tumor burden is the foundation of local control of the disease and contributes to improved efficacy of chemotherapy and radiotherapy. However, due to the rarity of orbital RMSs, there are few published studies on the clinical and pathological factors associated with orbital RMSs. The existed ones provide only limited information due to the small numbers of the cohorts or single-center nature of the studies. Moreover, the tumor-node-metastasis (TNM) staging system, which is the most common cancer staging system, features no established criteria for anatomic staging of RMSs or prognostic prediction (5). In addition, the TNM staging system includes only tumor size, lymph node (LN) metastasis, and distant metastasis, ignoring age, gender, tumor pathological type, and tumor grade. Therefore, the establishment of a specific staging system is important to stratify the prognosis of patients with orbital RMSs after surgery.

The development of a treatment paradigm has resulted in improved survival, with many studies reporting that 5-years overall survival (OS) rates varied from 75 to 90% (3, 6, 7). The improved prognosis and prolonged survival have led to a greater focus on the increasing rates of comorbidities. Previous studies indicated that deaths due to causes other than the primary cancer may account for a significant proportion of all mortalities of patients with cancers (8, 9). Previous research emphasized that failure to recognize the presence of competing risks may lead to misleading conclusions. Studies also highlighted that it was essential to consider competing risks when evaluating the prognosis, especially for patients with long-term survival (10, 11).

In this study, a competing risk analysis was conducted in patients with orbital RMSs after surgery using a large, population-based cancer dataset. We also established simple nomograms for predicting both the OS and cancer-specific survival (CSS) of orbital RMS patients after surgery.

## Materials and Methods

### Patient

The Surveillance, Epidemiology, and End Results (SEER) database was used in this study. Data on patients with orbital RMSs were extracted from the SEER database from 1973 to 2015. The following International Classification of Diseases for Oncology was adopted, Third Edition (ICD-O-3), histology code: 8,900, 8,901, 8,902, 8,910, 8,912, and 8,920; and the ICD-O-3 site code C69.6. The exclusion criteria were as follows: (1) patients with a second primary cancer, (2) patients not pathologically diagnosed, and (3) patients with missing or incomplete information on clinicopathological characteristics. Three-fourths of the patients were randomly selected to form a training cohort and to develop the nomograms, and the remaining patients were used as an internal validation cohort.

### Data Collection

The following variables were selected from SEER database in this study: age at diagnosis, gender, tumor size, site, grade, pathological type, tumor (T) stage (8th), LN metastasis, distant metastasis, race, and surgery.

### Statistical Analysis

Statistical analyses were conducted by R version 3.4.2 software (The R Foundation for Statistical Computing, Vienna, Austria. http://www.r-project.org). The OS was analyzed using Kaplan-Meier curves and compared with long-rank test. Fine and Grey's model were adopted to evaluate the cumulative incidence function (CIF) of the variables on cause-specific mortality (12, 13). Hazard ratio (HR) and the associated 95% confidence interval (CI) were calculated. A two tailed *P* < 0.05 was considered statistically significant.

Independent risk factors selected by the Cox regression and Fine and Grey's model were used to construct nomogram to predict OS and CSS, respectively. The main packages in this study were crprep and rms, which were used for construction of nomogram of CSS and OS. The predictive timepoints were set as 10, 20, and 40 years, respectively. Concordance index (C-index) and calibration curves (14, 15) were used to assess the discrimination and calibration power of the nomograms. Bootstraps with 1,000 resamples were used for the development of the nomogram and calibration curve to reduce the overfit bias and the area under receiver operating characteristic (ROC) curve (AUC) was used to evaluate the 10-, 20-, and 40-year, survival predictions.

## Results

### Characteristics of the Patients

In this study, data on 217 patients with orbital RMSs were retrospectively collected. The median age of the whole study cohort was 6 years. One hundred-nineteen (54.8%) patients were males, most of whom were white (*n* = 165, 76%). Nearly half the patients (*n* = 109, 50.2%) had underwent surgical resection. Another 22 patients were advised surgery but they did not receive surgical resection, and 86 patients were not recommended to receive surgery. There were 163 patients in the training cohort and 54 patients in the validation cohort. Table 1 shows a summary of the baseline clinicopathological characteristics of the patients. Eighty-three (50.9%) patients in the training cohort, and 22 (40.7%) patients in the validation cohort were older than 6 years old, and surgical resection was performed in 50% of the patients in both the training and validation cohorts. The clinicopathological factors of the patients in the training and validation cohort were similar.

**Table 1:** The comparison of clinicopathological factors between training cohort and validation cohort.

**Characteristic**		**N**	**Patients**		***P***
			**Training cohort**	**Validation cohort**	
Total		217	163	54	
Age (years)	≤6	112	80	32	0.212
	> 6	105	83	22	
Gender	Female	98	79	19	0.114
	Male	119	84	35	
Tumor size (cm)	≤2	47	36	11	0.851
	>2	170	127	43	
Tumor size (cm)	≤2	47	36	11	0.635
	2 ~ 4	120	92	28	
	> 4	50	35	15	
Tumor site	Left orbit	105	80	25	0.755
	Right orbit	112	83	29	
Tumor grade	Well	29	21	8	0.732
	Moderate	82	64	18	
	Poor	106	78	28	
Pathological type	Non-alveolar type	193	145	48	0.989
	Alveolar type	24	18	6	
T stage (8th)	T1	19	14	5	0.569
	T2	111	84	27	
	T3	58	46	12	
	T4	29	19	10	
LN metastasis	Absent	188	142	46	0.818
	Present	29	21	8	
Metastasis	Absent	200	149	51	0.517
	Present	17	14	3	
Race	Black	29	24	5	0.520
	White	165	121	44	
	Others	23	18	518	
Surgery	Performed	109	81	28	0.893
	Recommended, not performed	22	16	6	
	Not recommended	86	66	20	

*LN, lymph node*.

### Analysis of OS and CSS

**Figure 1:**
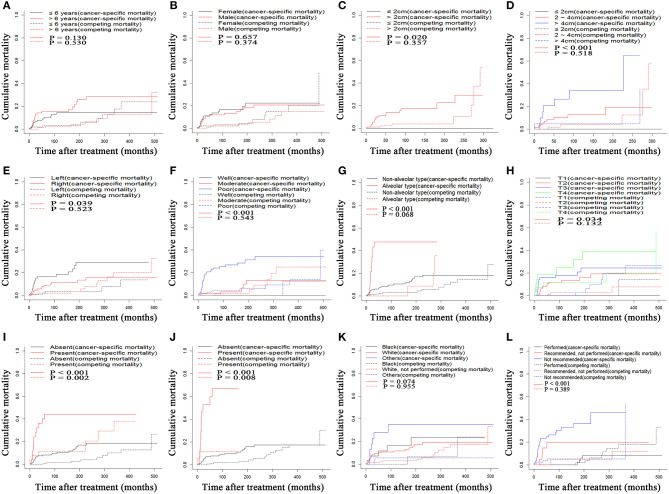
Cumulative cancer-specific and competing mortality according to patient characteristics: **(A)** Age, **(B)** Gender, **(C)** Tumor size, **(D)** Tumor size, **(E)** Tumor site, **(F)** Tumor grade, **(G)** Pathological type, **(H)** T stage (8th), **(I)** LN metastasis, **(J)** Metastasis, **(K)** Race, **(L)** Surgery. LN, lymph node.

During the follow-up period, 36 (22.1%) and 13 (24.1%) deaths occurred in the training cohort and validation cohort, respectively. In the training cohort, 27 (75.0%) deaths were the direct results of orbital RMSs, and 9 (25.0%) deaths were due to competing risk events. Seven cancer-specific deaths and six competing-risk deaths occurred in the validation cohort. The comparisons of 10-, 20-, and 40-years OS rates, cancer-specific mortality, and non-cancer-specific mortality are shown in Table 2. Patients older than 6 years old did not have higher rates of cancer-specific mortality. Tumor larger than 4 cm, located in the left orbit, and poorly differentiated were associated with higher cumulative incidences of cancer-specific mortalities. In addition, similar to OS, higher numbers of cancer-specific deaths occurred among patients with more advanced T stages (8th), LN metastasis, and distant metastasis. There were no significant differences in non-cancer-specific mortality stratified by these factors, except for the presence of LN and distant metastasis, which was associated with higher non-cancer-specific mortalities (Figure 1).

**Table 2:** Overall survival rates and cumulative incidences of mortality among patients with orbital RMS.

**Characteristic**		**Patients**		**Overall survival rate (%)**			***P***	**Cancer-specific mortality (%)**			***P***	**Non-cancer-specific mortality (%)**			***P***
		**No.**	**%**	**10-years**	**20-years**	**40-year**		**10-years**	**20-years**	**40-years**		**10-years**	**20-years**	**40-years**	
Total		217	100%	82.5	72.2	48.9		14.8	21.7	21.7		2.7	6.2	17.2	
Age (years)	≤6	112	52%	82.6	80.0	61.8	0.520	14.4	14.4	14.4	0.130	3.0	5.6	2.4	0.530
	> 6	105	48%	82.2	60.5	39.3		15.4	28.4	28.4		2.4	6.6	12.6	
Gender	Female	98	45%	81.1	69.9	28.6	0.352	16.8	22.3	22.3	0.657	2.1	7.8	2.1	0.374
	Male	119	55%	83.6	74.0	64.5		13.2	20.8	20.8		3.2	5.2	14.7	
Tumor size (cm)	≤2	47	22%	97.8	86.1	74.2	0.009	2.2	9.8	9.8	0.020	0.00	0.04	16.0	0.357
	>2	170	78%	77.9	67.9	38.4		18.6	25.4	25.4		3.5	6.7	17.1	
Tumor size (cm)	≤2	47	22%	97.8	86.1	74.2	<0.001	2.2	9.8	9.8	<0.001	0.00	4.1	16.0	0.518
	2~4	120	55%	86.9	76.6	32.9		10.8	17.1	17.1		2.2	6.3	17.1	
	> 4	50	23%	55.8	44.6	33.5		37.6	48.8	48.8		6.6	6.6	17.8	
Tumor site	Left orbit	105	48%	77.9	67.3	57.4	0.122	18.5	29.0	-	0.039	3.7	3.7	-	0.523
	Right orbit	112	52%	86.6	76.4	51.5		11.4	15.7	15.7		2.0	7.9	19.9	
Tumor grade	Well	29	13%	100.0	100.0	100.0	<0.001	0.00	0.00	0.00	<0.001	0.00	0.00	12.5	0.543
	Moderate	82	38%	95.1	77.7	61.7		3.1	13.3	13.3		1.7	9.0	24.9	
	Poor	106	49%	70.5	59.9	25.9		26.9	34.3	34.3		4.1	5.8	13.9	
Pathological type	Non-alveolar type	193	89%	0.864	0.759	0.538	<0.001	10.5	18.2	18.	<0.001	3.1	5.9	14.6	0.068
	Alveolar type	24	11%	0.524	0.437	0.164		47.6	47.6	-		0.0	8.7	-	
T stage (8th)	T1	19	09%	100.0	100.0	85.7	<0.001	0.0	0.0	0.0	0.034	0.0	0.0	14.3	0.132
	T2	111	51%	84.2	78.2	73		13.3	19.3	19.3		2.5	2.5	7.7	
	T3	58	27%	82	66	49.1		18.0	24.4	24.4		0.0	9.6	2.6	
	T4	29	13%	63.3	49.2	0		25.0	39.1	39.1		11.7	11.7	19.9	
LN metastasis	Absent	188	87%	88.1	77.7	55.1	<0.001	10.5	18.4	18.4	<0.001	1.4	3.9	12.7	0.002
	Present	29	13%	44.3	35.5	17.7		44.2	44.2	-		11.5	20.4	-	
Metastasis	Absent	200	92%	88.1	77.0	52.2	<0.001	10.1	17.3	17.3	<0.001	1.9	5.6	17.4	0.008
	Present	17	8%	21.2	21.2	21.2		67.1	-	-		11.8	-	-	
Race	Black	29	13%	83.5	70.1	52.6	0.141	16.5	23.5	-	0.074	0.0	6.4	-	0.955
	White	165	76%	85.5	74.6	47.7		11.7	19.3	19.3		2.8	6.1	17.1	
	Others	23	11%	59.6	59.6	59.6		34.9	34.9	34.9		5.5	5.5	5.5	
Surgery	Performed	109	50%	100.0	85.8	59.1	<0.001	0.00	8.1	8.1	<0.001	0.0	6.1	18.0	0.389
	Recommended, not performed	22	10%	75.3	75.2	68.4		19.8	19.8	-		5.0	5.1	-	
	Not recommended	86	40%	62.1	48.9	0		32.7	45.9	-		5.2	5.2	NA	

*RMS, rhabdomyosarcoma; other abbreviations as in Table 1*.

**Figure 2:**
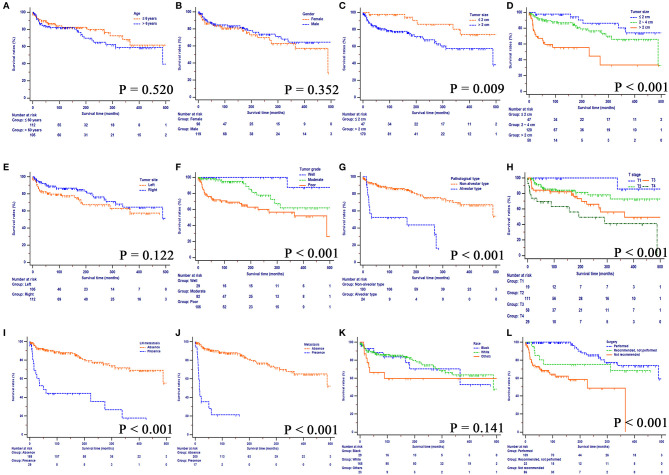
Overall survival rates according to patient characteristics: **(A)** Age, **(B)** Gender, **(C)** Tumor size, **(D)** Tumor size, **(E)** Tumor site, **(F)** Tumor grade, **(G)**, Pathological type, **(H)** T stage (8th), **(I)** LN metastasis, **(J)** Metastasis, **(K)** Race, **(L)** Surgery. LN, lymph node.

The median OS for the whole study cohort was 488.0 months. Fewer than 50% deaths were due to the orbital RSS. The median CSS was unavailable. The 10-, 20-, and 40-years OS rates were 82.5, 72.2, and 48.9%, respectively, and the 10-, 20-, and 40-years CSS rates were 85.0, 77.8, and 77.8%, respectively. Kaplan-Meier curves of OS, stratified by the clinicopathological factors are shown in Figure 2. Smaller tumors, well-differentiated tumors, non-alveolar pathological types, earlier T stages (8th), no LN or distant metastasis, and surgery contributed to improved OS of these patients. There was no significance in survival according to age, gender, tumor site and race.

### Construction and Validation of the Nomogram

**Figure 3:**
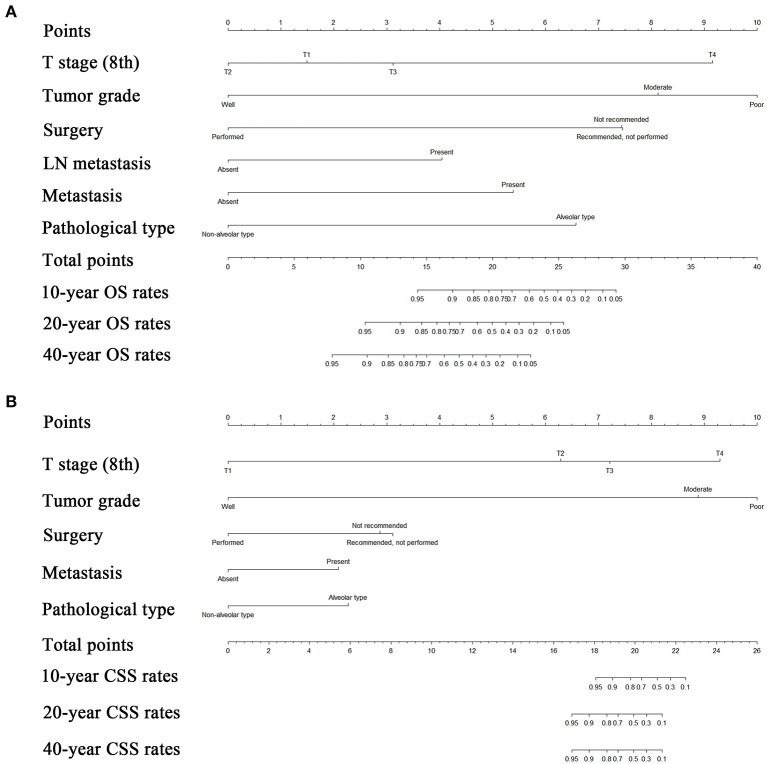
Nomograms predicting 10-, 20-, and 40-years OS **(A)** and CSS **(B)** of patients with orbital RMS. OS, overall survival; CSS, cancer-specific survival; LN, lymph node; RMS, rhabdomyosarcoma.

Risk factors were identified by univariate analyses, which indicated that tumor size, tumor grade, pathological type, T stage, LN metastasis, distant metastasis, and surgery were significantly associated with OS. All these variables were entered into a multivariate analyses, The results of this analysis showed that tumor grade (HR = 2.28, 95% CI = 1.30–4.00, *P* = 0.004), pathological type (HR = 3.51, 95% CI = 1.67–7.37, *P* = 0.001), T stage (HR = 2.72, 95% CI = 1.83–4.06, *P* < 0.001), LN metastasis (HR = 3.14, 95% CI = 1.51–6.50, *P* = 0.002), distant metastasis (HR = 3.11, 95% CI = 1.29–7.51, *P* = 0.012), and surgery (HR = 2.43, 95% CI = 1.58–3.74, *P* < 0.001) remained significant prognostic factors for OS (Table 3). The proportional subdistribution hazard assumption also held for variables in the CSS analysis. The results showed that poorly differentiated (HR = 3.34, 95% CI = 1.39–8.05, *P* = 0.007) or alveolar pathological type (HR = 2.93, 95% CI = 1.17–7.34, *P* = 0.022) tumor, more advanced T stage (8th) (HR = 3.20, 95% CI = 1.89–5.41, *P* < 0.001), presence of metastasis (HR = 3.74, 95% CI = 1.40–9.97, *P* = 0.008) and no surgical resection (HR = 2.80, 95% CI = 1.60–4.92, *P* < 0.001) all contributed to decreased CSS of patients with orbital RMSs. Nomograms were constructed for predicting both OS and CSS on the basis of these independent factors (Figure 3). In these two nomograms, the tumor grade and T stage (8th) contributed most to the prognoses of the patients, followed by surgery, pathological type of tumor, and metastasis.

**Table 3:** Univariate and multivariate analyses of survival in patients with orbital RMS.

**Characteristic**		**Overall survival**						**Cancer-specific survival**					
		**Univariate analysis**			**Multivariate analysis**			**Univariate analysis**			**Multivariate analysis**		
		**HR**	**95%CI**	***P***	**HR**	**95%CI**	***P***	**HR**	**95%CI**	***P***	**HR**	**95%CI**	***P***
Age (years)	<6/≥6	1.345	0.76–2.37	0.305			NI	1.68	0.84–3.35	0.142			NI
Gender	Female/Male	0.77	0.44–1.34	0.354			NI	0.85	0.43–1.66	0.624			NI
Tumor size (cm)	≤2/>2	2.98	1.26–7.03	0.013	1.35	0.36–5.01	0.653	3.73	1.14–12.22	0.030	1.71	0.32–9.14	0.532
Tumor size (cm)	≤2/2~4/> 4	2.94	1.87–4.62	<0.001	1.49	0.75–2.93	0.252	3.54	2.01–6.22	<0.001	1.65	0.74–3.68	0.218
Tumor site	Left orbit/Right orbit	0.64	0.36–1.13	0.126			NI	0.49	0.24–0.97	0.042	0.52	0.24–1.15	0.106
Tumor grade	Well/Moderate/Poor	3.20	1.83–5.57	<0.001	2.28	1.30–4.00	0.004	5.72	2.36–3.90	<0.001	3.34	1.39–8.05	0.007
Pathological type	Non–alveolar type/Alveolar type	4.87	2.57–9.24	<0.001	3.51	1.67–7.37	0.001	4.81	2.33–9.91	<0.001	2.93	1.17–7.34	0.022
T stage (8th)	T1/T2/T3/T4	1.96	1.41–2.73	<0.001	2.72	1.83–4.06	<0.001	1.87	1.25–2.79	0.002	3.20	1.89–5.41	<0.001
LN metastasis	Absent/Present	5.58	3.08–10.12	<0.001	3.14	1.51–6.50	0.002	4.72	2.30–9.71	<0.001	1.76	0.72–4.30	0.213
Metastasis	Absent/Present	15.07	7.39–30.72	<0.001	3.11	1.29–7.51	0.012	15.02	6.95–32.50	<0.001	3.74	1.40–9.97	0.008
Race	Black/White/Others	1.39	0.76–2.54	0.285			NI	1.60	0.79–3.25	0.191			NI
Surgery	Performed/Recommended, not performed/Not recommended	2.73	1.89–3.94	<0.001	2.43	1.58–3.74	<0.001	3.52	2.14–5.80	<0.001	2.80	1.60–4.92	<0.001

*HR, hazard ratio; CI, confidence interval; NI, not included; other abbreviations as in Table 1*.

**Figure 4:**
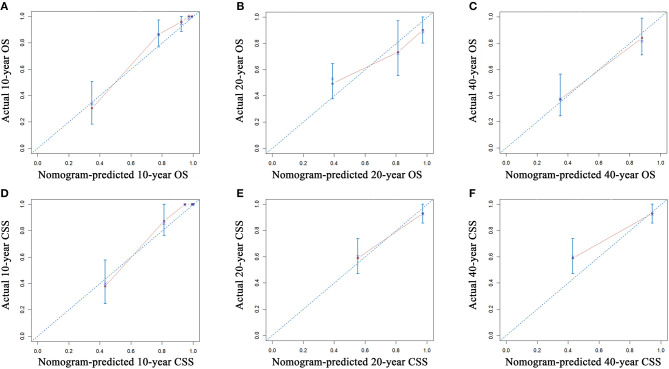
Calibration plots of the nomogram for 10-, 20-, and 40-years OS prediction **(A–C)** and CSS prediction **(D–F)**. The X-axis represents the nomogram-predicted probability of survival, and the Y-axis represents the actual OS probability. In a perfectly accurate nomogram, the prediction model would result in a plot where the observed and predicted probabilities for the given groups fall along the 45-degree line. Dots with bars represent nomogram-predicted probabilities, along with the 95% CI. OS, overall survival; CSS, cancer-specific survival; CI, confidence interval.

The established nomograms were used to estimate 10-, 20-, and 40-years OS and CSS rates by calculating the sum of points corresponding to the patient's characteristics. The accuracy of the nomogram in predicting OS was good, with a C-index of 0.901 (95% CI, 0.866–0.936) for the training cohort and 0.944 (95% CI, 0.907–0.981) for the validation cohort. The accuracy of the model for CSS prediction was also good, with a C-index of 0.923 (95% CI, 0.892–0.954) for the training cohort and a C-index of 0.904 (95% CI, 0.831–0.977) for the validation cohort, which suggested relatively good nomogram discriminative ability. The C-indexes of TNM for OS and CSS prediction were 0.702 and 0.688, respectively. Compared with TNM stage system, the established nomogram showed significantly higher predictive efficacy in survival prediction (*P* < 0.001). Calibration plots for survival prediction also showed fair agreement between predicted and actual survival (Figure 4). In addition, optimal predictive power of prognoses was indicated by the prognostic ROC analysis, which showed the values of the AUC for 10-, 20-, and 40-years OS and CSS prediction were 0.908, 0.826, and 0.847, respectively and 0.924, 0.863, and 0.863, respectively.

## Discussion

RMSs, which are considered the most common soft-tissue sarcomas of childhood, with the most frequent site of occurrence in the orbit, affect children more commonly (16, 17). Although the annual incidence of RMSs continues to rise steadily, the rarity of these types of tumors and the compressive retrospectively studies based on small numbers of patients make it difficult to evaluate the clinical characteristics of patients and to make an accurate estimation of survival outcomes. Moreover, the TNM staging system functions more as a clinical classification than prognosis-predicting system due to unavailability of a specific staging system for RMCs. As the patients with orbital RMSs in the present study had a relatively good prognosis, non-cancer-specific mortality need to be taken into account when evaluating the prognosis of this disease. In this study, we aimed to evaluate the prognostic factors and establish staging systems specifically for estimating OS and CSS of patients with orbital RMSs. The established nomograms were derived from a large cohort from the SEER database, showing favorable discrimination and calibration.

Surgical resection has long been an important component of the multiple treatment approach to orbital RMSs. In this study, the 10-, 20-, and 40-years OS rates of the patients after surgery were 100, 85.8, and 59.1%, respectively, which were significantly higher than those of patients who had not undergone resection. The results showed that patients who were not advised to undergo surgical resection were 5.72 times more likely to have a lower survival time as compared with that of patients who underwent surgical resection. Interestingly, patients who were not advised to undergo surgery were 2.10 times more likely to have poor survival than patients who did not receive surgical treatment, despite surgery being recommended. However, the established nomograms indicated that the contribution to poor survival was similar among all patients who did not undergo surgical resection, irrespective of whether a surgical approach was recommended. These results showed that surgery played an extremely important role in improving the prognosis of patients with orbital RMSs. The data in the present study also showed that the involvement of adjacent periorbital structures, such as the eyelids, conjunctiva, sinuses, and brain, precluded radical resection. Radiotherapy and chemotherapy were helpful for local control of disease after surgery. A multidisciplinary-based approach including surgery might offer the best chance of obtaining a favorable prognosis among patients with orbital RMSs (18, 19).

In common with other kinds of cancers (11, 20, 21), routine clinical and pathological factors in the TNM staging system, including the T stage (8th), LN metastasis, and distant metastasis, were associated with the prognosis of the patients. The established nomograms illustrated that the T stage (8th) had the biggest influence on survival. The results showed that there were 12.94, 6.72, and 3.72 times for T4, T3, and T2 stage patients, respectively, to have decreased survival as compared with patients with T1 stage. In the present study, tumor size, which is an important component of the T stage, was not established as an independent prognostic factor when it was analyzed together with the T stage in the multivariate analysis, indicating that apart from tumor size, the involvement of the periorbital structures might be a potential prognostic factor in patients. Thus, attention should be paid to the surgical removal and local reinforcement by radiation and chemotherapy of the periorbital involvement of disease. LN metastasis and distant metastasis are indications of advanced-stage disease and are regarded as contraindications for surgery. In this study, LN metastasis was an independent prognostic factor for OS, and distant metastasis was a significant predictor of worse OS and CSS of patients with orbital RMSs.

Apart from common pathological factors, our staging system indicated that the magnitude of a poor prognosis as tumor grade changed from well to poorly differentiated. Moreover, the pathological type was an independent prognostic factor among orbital RMS patients, and the alveolar type represented a significantly worse prognosis compared with other types of orbital RMSs in this study. This finding was in accordance with that of other reports (3, 4, 22). The tumor grade and pathological type were the intrinsic nature of the tumor and were independent of other pathological factors, such as tumor size, LN metastasis, or distant metastasis. According to the established nomograms, patients with different tumor grades or histological types would be assigned different scores indicating different survival times, irrespective of whether they were classified as the same TNM stage. In current classification systems, the tumor grade and pathological type are used to assess long-term survival (5). The additional inclusion of the tumor grade and pathological type in the proposed system partly explains the differences in the prognosis predicted by the TNM stage system and the nomograms established herein. The inclusion of these parameters could enhance the predictive power of nomograms in estimating the prognosis in patients with orbital RMSs.

In our study, causes other than the primary orbital RMS contributed to 30.6% of the deaths. As far as we know, this is the first study to build competing risk nomograms for patients with orbital RMSs. The established nomograms were well-calibrated and showed relatively strong predictive power. The C-indexes of nomograms for OS and CSS prediction in both the training and validation cohorts were all higher than 0.90, showing that there was a >90% probability that earlier death would be observed in individuals with a higher nomogram score than those with a lower score. The well-fitted calibration plots and relatively high values of the AUC also indicated the good accuracy of the established nomograms. Additional strengths of this study were the large cohort size and long-time follow-up period. As the first competing risk nomogram based on the largest cohort and longest time follow-up, the findings can be promoted in clinical practice where they can be used to predict survival rates in patients orbital patients RMSs after surgery.

This study has several limitations that should be noted. A major limitation of the present study was that the variables used to construct the nomograms represented only some clinical and pathological features. Data on various important tumor pathological features, including surgical margin status and tumor necrosis, were unavailable in the SEER dataset. We acknowledge that certain additional variables (e.g., pathological factors or molecular biomarkers) might provide potential predictive information. Similarly, the absence of information on radiotherapy and chemotherapy in the SEER database limits the use of the established nomograms to a certain degree. A lack of external validation was also a limitation of this study. Due to the rarity of this disease, it was difficult for the inclusion of adequate external validation in this study. Although the established nomograms exhibited good discrimination and validity, further validation based on an external cohort is needed to determine their validity.

In conclusion, we evaluated cancer-specific deaths and non-cancer-specific deaths in patients with orbital RMSs and built the first nomograms to specifically predict 10-, 20-, and 40-years OS and CSS using a large population-based cohort from the SEER database. The established nomograms can be used to provide accurate and valuable information for both patients and doctors, allowing tailored treatments for patients with orbital RMSs after surgery.

## Data Availability Statement

The raw data supporting the conclusions of this article will be made available by the authors, without undue reservation.

## Ethics Statement

This study was approved by the Institutional Review Board of Zhongshan Ophthalmic Center and Sun Yat-sen University Cancer Center. Written informed consent to participate in this study was provided by the participants' legal guardian/next of kin.

## Author Contributions

HX was responsible for conception, design, quality control of this study, and reviewed, and edited the manuscript, respectively. YZ and YL performed the study selection, data extraction, statistical analyses, and was major contributors in writing the manuscript. YZ and CH participated in studies selection and statistical analyses. YZ, CH, and HX contributed in classification criteria discussion. YZ and CH contributed to the writing of manuscript. All authors have read and approved the final version of the manuscript.

## Conflict of Interest

The authors declare that the research was conducted in the absence of any commercial or financial relationships that could be construed as a potential conflict of interest.

## References

[1] Pappo AS. Rhabdomyosarcoma and other soft tissue sarcomas in children. Curr Opinion Oncol. (1996) 8:311–6. 10.1097/00001622-199607000-00008 8869806

[2] Paulino A.C Okcu MF. Rhabdomyosarcoma. Curr Problems Cancer. (2008) 32:7–34. 10.1016/j.currproblcancer.2007.11.001 18206520

[3] Oberlin O Rey A Anderson J Carli M Raney R.B Treuner J. Treatment of orbital rhabdomyosarcoma: survival and late effects of treatment–results of an international workshop. J Clin Oncol. (2001) 19:197–204. 10.1200/JCO.2001.19.1.197 11134213

[4] Shields J.A Shields CL. Rhabdomyosarcoma: review for the ophthalmologist. Sur Ophthalmol. (2003) 48:39–57. 10.1016/S0039-6257(02)00415-0 12559326

[5] Amin MB Greene F AJCC Cancer Staging Manual, 8th ed Chicago, IL: Springer (2017).

[6] Arndt CA Stoner JA Hawkins DS Rodeberg DA Hayes-Jordan AA Paidas CN. Vincristine, actinomycin, and cyclophosphamide compared with vincristine, actinomycin, and cyclophosphamide alternating with vincristine, topotecan, and cyclophosphamide for intermediate-risk rhabdomyosarcoma: children's oncology group study D9803. J Clin Oncol. (2009) 27:5182–8. 10.1200/JCO.2009.22.3768 19770373 PMC2773476

[7] Raney R.B Walterhouse D.O Meza J.L Andrassy R.J Breneman J.C Crist W.M Results of the Intergroup Rhabdomyosarcoma Study Group D9602 protocol, using vincristine and dactinomycin with or without cyclophosphamide and radiation therapy, for newly diagnosed patients with low-risk embryonal rhabdomyosarcoma: a report from the Soft Tissue Sarcoma Committee of the Children's Oncology Group. J Clin Oncol. (2011) 29:1312–8. 10.1200/JCO.2010.30.4469 21357783 PMC3083999

[8] Schiergens TS Lindenthaler A Thomas MN Rentsch M Mittermeier L Brand K. Time-dependent impact of age and comorbidities on long-term overall survival after liver resection. Liver Int. (2016) 36:1340–50. 10.1111/liv.13068 26778517

[9] Szpakowski JL Tucker LY. Causes of death in patients with hepatitis B: a natural history cohort study in the United States. Hepatology. (2013) 58:21–30. 10.1002/hep.26110 23080403

[10] Zhou H Zhang Y Qiu Z Chen G Hong S Chen X. Nomogram to predict cause-specific mortality in patients with surgically resected stage i non-small-cell lung cancer: a competing risk analysis. Clin Lung Cancer. (2018) 19:e195–e203. 10.1016/j.cllc.2017.10.016 29153966

[11] He C Zhang Y Cai Z Lin X Li S. Overall survival and cancer-specific survival in patients with surgically resected pancreatic head adenocarcinoma: a competing risk nomogram analysis. J Cancer. (2018) 9:3156–67. 10.7150/jca.25494 30210639 PMC6134825

[12] Gray RJ A class of K-sample tests for comparing the cumulative incidence of a competing risk. Ann Stat. (1988) 16:1141–54. 10.1214/aos/1176350951

[13] Fine J.P Gray RJ A proportional hazards model for the subdistribution of a competing risk. J Am Statist Assoc. (1999) 94:496–509. 10.1080/01621459.1999.10474144

[14] Harrell FE Jr Lee K.L Mark DB. Multivariable prognostic models: issues in developing models, evaluating assumptions and adequacy, and measuring and reducing errors. Stat Med. (1996) 15:361–87. 10.1002/(SICI)1097-0258(19960229)15:4<361::AID-SIM168>3.0.CO;2-4 8668867

[15] Pencina MJ D'Agostino RB Overall C as a measure of discrimination in survival analysis: model specific population value and confidence interval estimation. Stat Med. (2004) 23:2109–23. 10.1002/sim.1802 15211606

[16] Andrade CR Takahama Junior A Nishimoto IN Kowalski LP Lopes MA. Rhabdomyosarcoma of the head and neck: a clinicopathological and immunohistochemical analysis of 29 cases. Brazil Dental J. (2010) 21:68–73. 10.1590/S0103-64402010000100011 20464324

[17] Eade E Tumuluri K Do H Rowe N Smith J. Visual outcomes and late complications in paediatric orbital rhabdomyosarcoma. Clin Exp Ophthalmol. (2017) 45:168–73. 10.1111/ceo.12809 27473389

[18] Schoot RA Saeed P Freling NJ Blank LE Pieters BR van der Grient JN. Local resection and brachytherapy for primary orbital rhabdomyosarcoma: outcome and failure pattern analysis. Ophthal Plast Reconstr Surg. (2016) 32:354–60. 10.1097/IOP.0000000000000562 26398242

[19] Blank LE Koedooder K van der Grient HN Wolffs NA van de Kar M Merks JH. Brachytherapy as part of the multidisciplinary treatment of childhood rhabdomyosarcomas of the orbit. Int J Rad Oncol Biol Phys. (2010) 77:1463–69. 10.1016/j.ijrobp.2009.06.015 19864080

[20] He C Mao Y Wang J Huang X Lin X Li S. Surgical management of periampullary adenocarcinoma: defining an optimal prognostic lymph node stratification schema. J Cancer. (2018) 9:1667–79. 10.7150/jca.24109 29760806 PMC5950597

[21] He C Mao Y Wang J Song Y Huang X Lin X. The predictive value of staging systems and inflammation scores for patients with combined hepatocellular cholangiocarcinoma after surgical resection: a retrospective study. J Gastroint Surg. (2018) 22:1239–50. 10.1007/s11605-018-3756-3 29667093

[22] Newton WA Jr Gehan EA Webber BL Marsden HB van Unnik AJ Hamoudi AB. Classification of rhabdomyosarcomas and related sarcomas. Pathol aspects and proposal for a new classification–an Intergroup Rhabdomyosarcoma Study. Cancer. (1995) 76:1073–85. 10.1002/1097-0142(19950915)76:6<1073::aid-cncr2820760624>3.0.co;2-l 8625211

